# Work of the Port of London Sanitary Committee

**Published:** 1921-08-06

**Authors:** 


### A PUBLIC SAFEGUARD.

### Work of the Port of London Sanitary Committee.

It is doubtful whether the majority of people, even those engaged in medical spheres, realise the enormous amount of work necessary to protect this country against invasion by disease in many of its most dangerous forms. If we take the instance of London alone the facts and figures are striking enough. When we appreciate that this is but one small part of the whole system the public debt to the sanitary services is both obvious and startling. Needless to say—for the results speak for themselves—this protective work is carried out with remarkable success. The jurisdiction of the Port of London Sanitary Committee extends from Teddington to the Nore, and although the duties carried out in any particular area may be characteristically constant and routine in nature, yet the supervision and co-ordination of the whole implies a striking all-round efficiency in the service.

### Colonial Mutton.

Food inspection may seem at first sight a relatively simple process. Perhaps it is as conducted before a students' class or in the ordinary circumstances of the retail trade. But when one is dealing with a vast mass of material such as may be found at the docks and warehouses it is indeed another matter. At a recent inspection there were at one of the great refrigerating sheds, namely, that of the Royal Albert Dock, in one block alone 250,000 carcases of mutton, and in another 288,000 cases of Colonial butter. Also, it is not alone the quality of this food as it arrives at these stores which must be watched. There is always the risk of damage and infection by vermin. The rat menace, for example, is one that has constantly to be combated. Many of the great sheds are now made rat-proof. Gradually more and more are being improved and brought up to standard, but the size of the campaign involved is colossal. In essential fact, the only way to subdue the rat is to prevent him from getting at any food. To trace the consequences further, the complete elimination of the plague menace will only come with the disappearance of the rat. And so might one proceed through a host of more complicated and less well-known causes and disease effects connected with food storage and importation. One of the most valuable indirect effects of this constant supervision is that produced in foreign countries, which are gradually realising that only sound material can be profitably sent into this country. The recent endeavour to raise, internationally, the standard of all food inspection and control is fortunately meeting with slow but steady response, and undoubtedly the honour of practically showing the way rests jointly with our own authorities and those of America. In any so-called political cries for economy the public will be well advised not to include this branch of sanitary work in their call for reductions of *personnel* and expenditure.

Perhaps one of the most illustrative examples of the magnitude of the work is seen in the lists of quantities of food actually destroyed in the Port of London as unfit for human consumption. Had even a portion of it escaped into our shops the result is better imagined than described. In 1920 over 400 tons of meat were so disposed of, together with 2,675 tons of fruit and game. Nearly 700 tons of general provisions and 131 tons of rabbits were similarly prevented from reaching our tables. The total weight of food condemned and "destroyed" for that year was well over 4,000 tons.

### Economy in Destruction.

But although one uses the term "destroyed," it must be remembered that all this material, though unsuitable and unsafe for the human being, can be satisfactorily treated to serve a variety of other uses. And here, therefore, arises another branch of this complicated scheme. Always the condemned goods must if possible be disposed of still to serve some useful purpose. Sterilisation by heat, complete cooking, and a variety of trade processes can be brought into play. Out of the 4,000 tons already mentioned only some 900 were actually incinerated and completely destroyed! Among the rest we see that 575 tons were boiled down to recover the fat and other animal by-products. Nearly 2,000 tons were suitable for conversion into prepared cattle and poultry foods. Also by carefully supervised processes of manufacture 450 tons was ultimately converted or partially converted into substances safe for human consumption, in the form of confectionery, etc. And there were some 130 tons of otherwise discardable material still found capable of giving useful manufacturing products when submitted to distillate processes. Therefore can one realise that the watch of the Committee does not end with the simple condemnation of a cargo or the contents of a warehouse. All these products and "foods" have to be traced, marked down in their movements, and their ultimate fate carefully certified by local authority.

A comparison of the quantities of food thus dealt with in various years is instructive.

| | Tons | | Tons |
|---|---|---|---|
| 1911 ... ... ... | 1,408 | 1916 ... ... ... | 5,664 |
| 1912 ... ... ... | 3,758 | 1917 ... ... ... | 4,847 |
| 1913 ... ... ... | 2,458 | 1918 ... ... ... | 1,200 |
| 1914 ... ... ... | 1,184 | 1919 ... ... ... | 2,829 |
| 1915 ... ... ... | 3,118 | 1920 ... ... ... | 4,102 |

As the war period is covered it is difficult to draw direct deductions from the fluctuations which have occurred, but it will be seen that the quantity condemned or "destroyed" shows little tendency actually to decrease, although if considered as a percentage of all cargoes it might probably do so. The most impressive fact remains, therefore, that there is urgent need for this public service, for its support, and for its increase as and whenever necessity may require.

